# Systemic Neoplastic Cryoglobulinemic Vasculitis Mimics Large Vessel Occlusion: A Case Report

**DOI:** 10.1097/NRL.0000000000000489

**Published:** 2023-03-06

**Authors:** Antonio L. Bisogno, Federica Viaro, Alessio Pieroni, Francesca Rinaldi, Maurizio Corbetta, Claudio Baracchini

**Affiliations:** * Department of Neuroscience; † Padova Neuroscience Center (PNC), University of Padova; ‡ University Hospital of Padova; § Venetian Institute of Molecular Medicine, Padova, Italy

**Keywords:** stroke, vasculitis, large vessel occlusion, thrombectomy

## Abstract

**Introduction:**

We describe a systemic neoplastic cryoglobulinemic vasculitis presenting as a large vessel occlusion (LVO) syndrome. We focus on a rare presentation of a rare condition.

**Case Report:**

A 68-year-old man was admitted to the Stroke Unit of Padova with a right middle cerebral artery syndrome. A cerebrovascular event was suspected and protocol for revascularization treatment was performed. Neuroimaging provided no evidence for infarcted tissue or medium-large vascular occlusion but hypothesized a vasculitic involvement of the small vessels of the right hemisphere. Further diagnostics demonstrated a microangiopathic involvement of the heart, kidneys, and lungs. Blood tests showed circulating cryoglobulins and further hematological investigation identified a chronic lymphatic leukemia-like lymphoproliferative disorder. High-dose steroid therapy improved the patient’s clinical status and no neurological symptoms remained at discharge.

**Conclusion:**

We discuss the clinical-radiologic presentation of a small vessel vasculitis that mimics an LVO stroke. This case focuses on the relevance of concomitant multiorgan manifestations in the hyper-acute evaluation of LVO stroke, suggesting the clinical neurologist should consider alternative etiologies as these could provide important clinical implications.

We report a case of a large vessel occlusion (LVO) stroke syndrome, which ultimately was diagnosed as systemic neoplastic cryoglobulinemic vasculitis. Cryoglobulinemic vasculitis typically affects small vessels of the body and is usually associated with connective tissue diseases, infections, and lymphoproliferative disorders. Neurological involvement includes encephalopathic symptoms, motor/sensory peripheral neuropathy, and only rarely focal neurological signs.^1–3^ Although neurological manifestations are usually described in patients with connective tissue diseases or infections such as hepatitis *C*,^1^ not much is known regarding the central nervous system (CNS) presentation of neoplastic cryoglobulinemia as described in this case.

## CLINICAL CASE

**FIGURE 1:**
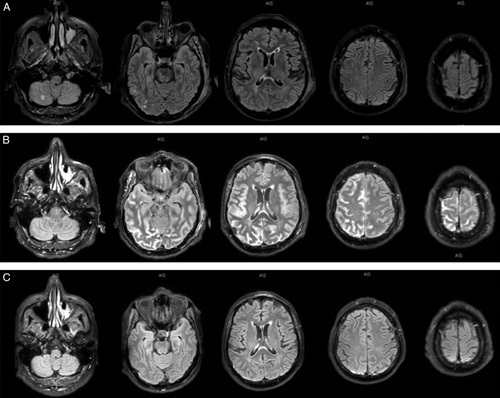
Fluid attenuated inversion recovery scans. (A) Magnetic resonance imaging (MRI) performed on day 1 showed punctate lesions in the cerebellum bilaterally and a small right parieto-temporal hematoma. (B) MRI performed on day 2 showed (in addition to the parietal hematoma) diffuse hyperintensity of the sulcal regions of the right hemisphere, left posterior parietal and fronto-mesial regions, and a linear right pachymeningeal enhancement (C) magnetic resonance performed on day 14 showed a slight right posterior pachimeningeal enhancement.

A 68-year-old man with hypertension and diabetes type II with a recent negative evaluation for recurrent abdominal pain presented to the emergency room for a right middle cerebral artery syndrome, last seen well 2 hours prior. He presented mild frontal and occipital excoriations and his NIHSS score was 18 for forced right gaze deviation, left hemianopia, dysarthria, left hemiplegia, and hemi-neglect.^4^ A cerebral computed tomography showed a mild posttraumatic right acute subdural parietal hematoma that contraindicated intravenous thrombolysis, whereas a cerebral angio-computed tomography scan excluded an LVO. A brain magnetic resonance imaging (MRI) with angio-magnetic resonance disclosed punctate lesions in the right parietal lobe and cerebellum bilaterally (Fig. 1A). A cerebral angiogram showed small vessels with an irregular appearance in the right parieto-occipital and left parietal regions, consistent with a vasculitic involvement, while no vasospasm was detected. The patient was admitted to the Stroke Unit of Padua University Hospital with a stable neurological status (NIHSS = 16), febrile, and high arterial blood pressure (>200/100 mm Hg). The EKG disclosed signs of acute subepicardic necrosis and a chest x-ray showed bilateral interstitial pulmonary infiltrates. No signs of endocarditis were found by a bedside transthoracic echocardiography. On day 2, the patient underwent a new MRI scan (Fig. 1B) that showed a fluid-attenuated inversion recovery hyperintensity in the sulcal regions of the right hemisphere, left posterior parietal regions, and a pachymeningeal enhancement after contrast agent infusion. Blood investigations identified hypoalbuminemia, acute renal failure, lowering Troponin I and brain natriuretic peptide levels, hyperglycemia, and slight anemia. cererbo-spinal fluid examination (including appearance, proteins, white cell count, red cell count, glucose, gram stain, microbiological film array, extended culture, and cytology) was normal. Due to these findings, sepsis, the acute appearance of purpura in the proximal region of the legs, we began empiric antibiotic therapy with levofloxacin. Further immunologic work-up included negative antineutrophil cytoplasmic antibodies, antinuclear antibodies, anti–double-stranded DNA antibodies, extractable nuclear antigens, and high levels of circulating rheumatoid factor. Additional testing found no anticitrullinated protein antibodies, reduced complement components, and circulating cryoglobulins (4% at cryocrit). In light of a concomitant CNS, renal, skin, cardiac, and pulmonary involvement (Table 1), we suspected a systemic small vessel vasculitis and investigated the possible origin of cryoglobulinemia. Microbiological investigations, including a test for hepatitis *C* virus, were negative, whereas the lymphocytic immune-phenotype and medullar biopsy showed clones of B cells expressing markers of chronic lymphatic leukemia (like). We started intravenous high-dose steroid therapy, with rapid improvement of vital parameters and normalization of the neurological examination on day 7. Blood tests showed a recovery of renal function and a control chest x-ray confirmed a complete resolution of the described pulmonary infiltrates. At 2 weeks from onset, a brain MRI showed a near complete resolution of the fluid-attenuated inversion recovery hyperintensities (Fig. 1C). The patient was discharged and followed up at our Day Hospital where he started a Rituximab-based therapy.

**TABLE 1:** Systemic Involvement on Hospital Admission (Central Nervous System excluded)

Organ	Symptoms	Blood tests	Other diagnostics
Kidney	Anasarca	Hypoalbuminemia, elevated creatinine, and urea levels	Proteinuria
Lung	Dyspnea, cough, and fever	Elevated CRP and pro-Calcitonine levels	Bilateral pulmonary infiltrates
Skin	Lower limbs purpura	Anemia, reduced complement components, high ESR levels	—
Heart	Tachycardia	Elevated TnI and BNP levels	EKG and bedside echocardiogram signs of necrosis

BNP indicates brain natriuretic peptide; CRP, C-reactive protein; ESR, erythrocyte sedimentation rate; TnI, troponin I.

## DISCUSSION

Cryoglobulins are serum proteins that precipitate at 4 °C and can be responsible for small vessel vasculitic syndromes. Although monoclonal cryoglobulins are commonly associated with lymphoproliferative disorders, mixed cryoglobulins are frequently detected in patients with connective tissue diseases or infections such as hepatitis *C*.^1^ Neurological manifestations are usually described in HCV cryoglobulinemia whereas not much is known about the CNS presentation of cryoglobulinemic vasculitis associated with lymphoproliferative disorders. In addition, although a stroke-like onset is associated with medium-large vessel vasculitis,^5,6^ small vessel involvement typically relates to general encephalopathic symptoms or motor/sensory peripheral neuropathy.^1–3^ In this report, we describe the clinical and radiologic presentation of a systemic neoplastic cryoglobulinemic vasculitis presenting as LVO stroke syndrome. We hypothesize that the involvement of broad regions of common vascular territories can mimic LVOs (Fig. 1B). In fact, after appropriate therapy and complete neurological recovery, MRI scans showed a significantly improved radiologic status (Fig. 1C), implying symptoms derived from local inflammation/blood flow alterations and only partially from tissue necrosis. We have focused on the clinical relevance of general systemic involvement in the evaluation of stroke syndromes (Table 1). In addition to being often associated with a stroke mimic,^7^ multiorgan manifestations in the hyperacute phase of LVO stroke should suggest alternative etiologies that might provide important therapeutic and prognostic implications.
